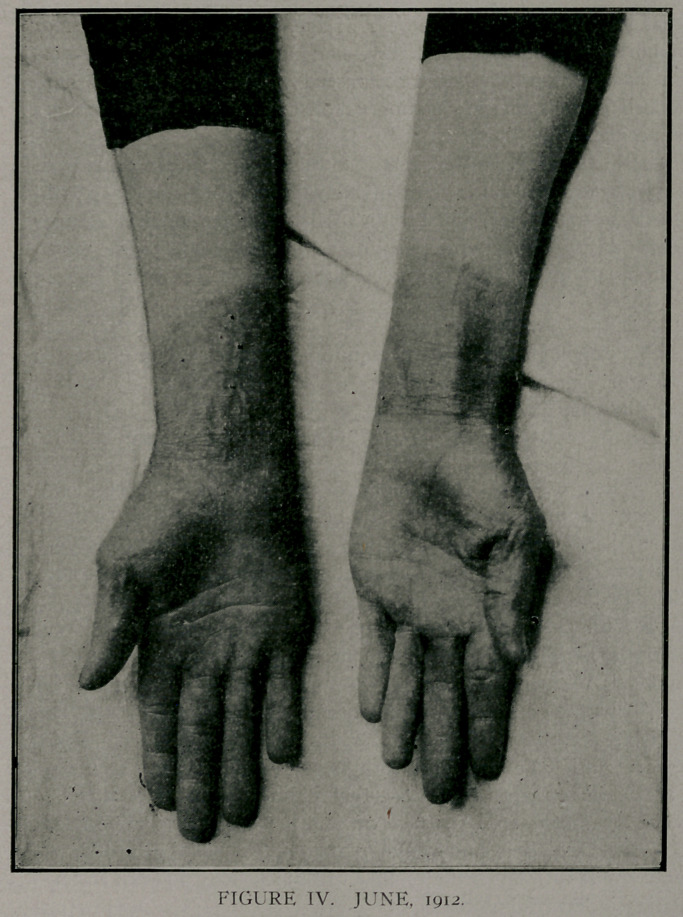# Etiologic Factors and Recurrent Attacks of Pellagra*Read before Second Triennial Meeting of the National Association for the Study of Pellagra Columbia. S. C. October 3 and 4, 1912.

**Published:** 1912-10

**Authors:** Geo. C. Mizell

**Affiliations:** Atlanta, Ga.


### ETIOLOGIC FACTORS AND RECURRENT ATTACKS OF PELLAGRA*

By Geo. C. Mizell, M.D., Atlanta, Ga.

At the present time a diagnosis of Pellargra is based wholly upon clinical features. These clinical features are not always constant, hence in the state of our present knowledge it is often difficult to say where the pellagrous process begins or where it ends. In view of these facts the term, recurrent attacks, used in this paper calls for some qualification. It appears to be the custom to consider the dermatitis as the one characteristic symptom, and if error is to be avoided a diagnosis should not be made without its presence.

In observing the course of the disease, it would be fair to presume that a case going through the summer in fair health, without developing the dermatitis when exposed to the sun, is no longer a pellagrin. Likewise it is to be concluded that a case developing the dermatitis under the same conditions is a pellagrin.

Because of the diagnostic significance of the dermatitis this symptom is used as an index to the state of the patients, i. e., when a given case has had an annual recurrence of the dermatitis, this is termed a recurrent attack of the disease or if a case goes through the year without dermatitis, it is held that he has not had a recurrence.

In doing this we will not lose sight of the probability that Pellagra is a chronic disease, characterized by peculiar exacerbations due to climatic and other influences.

Thus, in speaking of attacks, we mean an incident which always denotes the presence of the disease, yet there are probably cases in which this characteristic lesion never occurs.

The etiologic features that will be discussed are corn and cottonseed oil consumption, and incidentally age, sex, residence and season. These factors as they appear in one hundred cases will be the basis for our remarks.

By referring to the table it will be observed that the oil and lard consumers who had no attack in 1912 are placed first. Be-

* Read before Second Triennial Meeting of the National Association for the Study of Pellagra. Columbia. S. C. October 3 and 4, 1912.

low these are placed the cases who had an attack in 1912, except case number one hundred, which is placed alone at the bottom of the table.

After making this division, the cases are further divided into groups according to the consumption of corn and cottonseed oil.

Group I is composed of thirty-three cases, who until 1912 had been having attacks of pellagra for one, two, three or more years. Notwithstanding the fact that they ate corn products continuously, they did not have an attack in 1912. It is also to be noted that these patients, with few exceptions, enjoyed good health in 1912.

In Group II we have twenty-six cases who had discontinued corn consumption for some months and had no recurrence in 1912. It will be pointed out later that both of these groups, consisting of fifty-nine cases, together with three cases of Group III, made important changes in diet other than changes in corn consumption.

In Group III are six cases who never ate corn products. These six cases have had a total number of twelve attacks The first three made changes in their diet at the time of the first attack and did not have a recurrence. The last three did not make any changes and continued to have recurrent attacks. Case number sixty-three had attacks for five successive years.

The cases of Group IV, sixteen in number, suspended corn products and had a recurrense one or two years thereafter.

Group V, composed of eighteen cases, continued the use of corn products and have had a recurrence every year since the first attack.

Summing up the relationship of corn consumption to recurrent attacks, we have:-

| | |
|---|---|
| 51 cases continued corn. | 33 had no attacks in 1912. |
| | 18 had attacks, " " |
| 42 cases discontinued corn. | 26 had no attacks, in 1912. |
| | 16 had attacks, " " |

7 cases ate no corn. Had a total of 13 attacks.

The table shows that all of the one hundred cases were consumers of cottonseed oil products, except case number 100, Some effort has been made to show how long the oil had been consumed when the first attack occurred. The figures in the table represent the number of years the patients stated that they had been using this food when they developed pellagra. Where this time could not be fixed the number of years was left blank. The anual consumption in some cases, however, is limited to three four or five months of the year.

In Group VI are sixty-two cases who did not have a recurrence in 1912. Three cases had none in 1911. The members of this group had all suspended oil consumption some months before the Spring of 1912.

Group VII is composed of twenty-four cases who continued to consume oil products and continued to have recurrent attacks.

Group VIII is composed of 13 cases who had discontinued oil consumption and had a recurrence some months thereafter.

Group IX is composed of one case, a child, eight months old, who was not a consumer of either oil or corn.

Summing up the relationship of oil consumption to recurrent attacks, we have:-

| | |
|---|---|
| 24 cases continued oil | 24 cases had attacks in 1912.<br>0 cases had no attacks in 1912. |
| 75 cases discontinued oil | 62 cases had no attacks in 1912.<br>13 cases had attacks in 1912. |

One case ate no oil. Had an attack in 1911, none in 1912.

So far we have only stated the facts pertaining to oil and corn concumption in connection with recurrent attacks in one hundred cases of pellagra. We could have increased the number of cases to several hundred, but we believe the ones detailed are representative, and think we have a sufficiently large number to warrant some acurate observations and correct conclusions. We believe that a correct interpretation of the facts set forth in these cases will reveal the relationship of the factors mentioned to the etiology of the disease.

FIGURE I. FEBRUARY 7, 1912.

The maize theory has met with all but general acceptance, yet there remains obstacles which this conception of the etiology has not set aside. The most important of these objections, represented in the incidence of pellagra in individuals who do not eat corn and the continuation or cessation of attacks irrespective of the suspended or continued use of corn in the diet of pellagrins, is sufficient to refute any exclusive maize theory. Of the one hundred cases, seven were not corn consumers; five ate home-raised corn only. Case No. 48 selected his own home raised corn, supervised the nubbing and had it ground in his own mill, yet he had a most severe attack of pellagra, notwithstanding the fact that he ate very little of corn.

Our position in regard to this factor is based upon such observations and may be stated as follows: Corn, by virtue of the high percentage of fat and the nature of this fat, may possibly be capable of producing pellagra if consumed for a long time to the exclusion of fats. It is probably true that both sound corn and spoiled corn are equally effective in producing the disease, except that in the case of spoiled corn there are present other injurious products which predispose to an attack of illness through their effect on the general health. The facts set forth in the table show that corn as it is consumed in Georgia, South Carolina and Alabama, has no etiologic connection with pellagra. If it is consumed in sufficient quantity anywhere, either sound or spoiled, the fact remains that the specific agent has not been identified, and that it is not the only cause. Sound and spoiled corn should be withheld from the pellagrin because it contains a fat is injurious to him while he is so affected.

Before entering into the discussion of the several facts setting forth the relationship of cottonseed oil to pellagra, it will aid us in interpreting these facts to state the theory of the connection.

It is proposed, (1st) that when linolin (the characteristic fat of the cottonseed oil group) predominates in the fatty food of an individual it is deposited in the tissues as linolin and as linolyl compounds;

(2nd). That linolin and linolyl compounds by virtue of their unstable nature, readily undergo oxidation in marked contrast to

FIGURE II. FEBRUARY 7, 1912.

olein, palmitin and stearin, which are among the most stable organic compounds in the animal body.

(3rd). That fat and fatty tissue containing linolin and linolyl compounds in excess are unfit for performing the functions designed for normal animal fatty tissue.

(4th). That the end products of oxidation in the case of linolin are different from those of olein, palmitin and stearin.

Of course cottonseed oil can only be connected historically with the present day pellagra. It will not account for the disease two hundred years ago, but there is reason to believe that oils, or seed containing oil, of the same class have been eaten for centuries in pellagrous regions.

A partial list of the suspected oils is given below :-

### Oils Containing Linolin in Notable Per Cent.

#### PUMPKIN SEED OIL.

French—Huile de courge, huile de potiron.
German—Kurbissaninol, Kurbiskernol.
Italian—Olio di pucac.

#### MAISE OIL, CORN OIL.

French—Huile de maise.
German—Maisol.
Italian—Olio de mais, Olio di granturco.

#### KAPOK OIL.

French—Huile de kapok.
German—Kapokol.
Italian—Olio di kapok.

#### COTTON SEED OIL.

French—Hnile de coton.
German—Baumwollsamenol Cottonol.
Italian—Olio di cotone.

#### SESAME OIL, BENISEED OIL, GINGELLI OIL, TEEL OIL.

French—Huile de sesame.
German—Sesaaamol.
Italian—Olio di sesamo.

#### BRAZIL NUT OIL.

French—Huile de noix de Braasil.
German—Paranussal.
Italian—Olio di noci del Brasile.

POPPY SEED OIL.

French—Huile d'oeillette, Huile de pavot.
German—Mohaul.
Italian—Olio di papavero.

SUNFLOWER OIL.

French—Hiule de tournesol.
German—Souneufluemerol.
Italian—Olio di girasole.

WALNUT OIL, NUT OIL.

French—Huile de noix.
German—Nussol, Wallunssol.
Italian—Olio di noce.

FIGURE III. JUNE, 1912.

The only point that can be raised in opposition to either of the four propositions is in regard to the adaptability of linolin as a substitute for olein, palmitin and stearin in animal tissue. The difference in the chemical and physical properties of these fats seems to settle this question.

Bear in mind that this theory does not postulate the action of the neutral fat linolin as being a poisonous or toxic agent upon its entrance into the body but that it gradually accumulates in the absence of other fatty food and when it is in excess in the fatty tissues and fatty compounds it renders these tissues and compounds unstable.

Nothing outside of our observation has been found upon which to base a conclusion as to just what period of time is required for this transition, but evidently this period is measured by years. Certain tissues undoubtedly retain their constituents longer than others. For example, it is reasonable to suppose that the residence of fat in adipose tissue is of longer duration than fat in the skin. Certain individuals may use up their fatty tissue and replace it more rapidly than others. The state of an individual's health also plays a part in the destructive and constructive metamorphosis of this tissue.

It appears that it is necessary for oil to be consumed for not less than a year and when the time has been so short there is always a history of excessive use. These patients have used the fat exclusively and in many cases have seasoned boiled vegetables with it and have eaten much of fried foods. Almost invariably the pellagrin admits that he eats more greasy food than any other member of the family. This is especially true when the pellagrin and the other members of his family have previously enjoyed equal health.

In getting the history of oil consumption, the past eighteen months should be covered. Much difficulty is occasionally experienced, owing to the ignorance of some as to what they are eating. An intelligent patient states that he raises his own hogs and renders his own lard; denies visiting any where for any length of time. His wife supports these statements, yet it is finally shown that he has bought hogless lard for eighteen months prior to his first attack. An intelligent grocer states that he sends

FIGURE IV. JUNE, 1912.

the lard from his own store, and never uses anything but pure lard. Upon request he exhibits six empty buckets, only one of which is labelled "lard".

One significant point that may be mentioned here is that except in children under one year of age, all pellagrins, about four hundred in number, that I have had the opportunity of making an investigation, have been cottonseed oil consumers. It is hard to believe that this association of oil consumption with pellagra is accidental.

We have recognized the fact that many people have consumed cottonseed oil in some of the various forms for years without apparent injury, and this has led to inquiry among our patients not suffering with pellagra as to how many were eating cottonseed oil and how long they were using it.

*The results were as follows:-*

Forty-eight per cent. were users of cottonseed oil products;

Eighty per cent. of them date the beginning of its use within the past three years;

Many of these patients showed symptoms of Pellagra.

When we admit that the fat of the body has long residence therein, requiring months for any given area of fat to be utilized and replaced from the food which enters the body; that adipose tissue may remain intact for months, or even years, it becomes clear that the pellagrin will be liable to attacks just so long as his adipose tissue contains linolin. Also, that by marked reduction of the adipose tissue he will become immune to attacks until the linolin is reintroduced into his body.

Thus the period of accumulation or eradication becomes very variable according to the diet, health and individual disposition towards fat. We find such variations among our cases.

There is reason to believe that if linolin does not exceed a limited per cent. in a mixture of fats it will, on account of the ease with which it undergoes oxidation, be utilized before the more stable fat, thus being removed from the body and not accumulating in harmful amounts.

Just here it is interesting to note the physiological law which governs the deposit of fats. This law and this theory explain why the dermatitis is always more severe on the extremities and face when all parts are equally exposed to sunlight. Physiological compensation probably operates to reabsorb and transport the more liquid fats to the extremities, thus explaining the recurrence of dermatitis in these parts as long as three to twelve months after a previous attack, even though linolin has been excluded from the diet.

We were able to fix the number of years of oil consumption in seventy-three of these cases, as follows:

| | | | | | | | | | |
|---|---|---|---|---|---|---|---|---|---|
| 4 | had | eaten | cottonseed | products | 1 | year | before | first | attack; |
| 10 | " | " | " | " | 2 | " | " | " | " |
| 16 | " | " | " | " | 3 | " | " | " | " |
| 12 | " | " | " | " | 4 | " | " | " | " |
| 10 | " | " | " | " | 5 | " | " | " | " |
| 10 | " | " | " | " | 6 | " | " | " | " |
| 2 | " | " | " | " | 7 | " | " | " | " |
| 6 | " | " | " | " | 8 | " | " | " | " |
| 2 | " | " | " | " | 9 | " | " | " | " |
| 1 | " | " | " | " | 10 | " | " | " | " |

In Group VII we have twenty-four cases who used cottonseed oil continuously, or until a few months of the succeeding hot season. The cases who continued to eat oil products up to the time of the attack, or through the previous fall, had an equally severe recurrences. Cases Nos. 63, 80, 89, 91, and 94 represent a class of cases that have never had a severe attack. These mild cases always give a history of either interrupted oil consumption very little oil consumption, consumption of oil in the fall of the year only, or oil consumption for a short period of time. A case not included in the table gave a history of eating compound lard in the fall of the year for twenty-two years, and of recurrent attacks of pellagra eighteen years.

Cases Nos. 94, 83 and 56, consuming oil in this manner, had mild attacks annually for twelve, eight and four years respectively, without any serious impression of the malady.

In the table we find sixty-two people who discontinued the use of oil and had no attacks in 1912. Two, Nos. 22 and 60, had no attacks in 1911. All of these people discontinued the use of oil in August or earlier except Nos. 1, 2 and 33. These three were on a diet in the summer and fall of 1911 which excluded this food product and during the winter ate very little greasy food.

The cases of Group VIII, it is to be noted, had discarded oil several months but had a recurrence in 1912, though with one exception these patients were in much-improved or good health. They had no symptoms except a slight dermatitis. The one exception, No. 67, continued the use of oil through November, 1911 and suffered a severe attack in April 1912; he has made a good recovery, and in September was in good health.

We have already mentioned the possibility of the long residence of fat in the body and that as the more liquid fat of the extremities and head are oxidized they may be replaced by the more liquid fat which had been deposited near the warmer parts of the body.

So we would expect those cases who had suspended consumption of this class of fats to have an attack of dermatitis even after several months if exposed to the exciting cause. Illustrating this point is case No. 71. This patient suspended oil consumption in October, 1911, and on December 16, 1911, she had her first attack. The dermatitis affected the area which shows normal in Fig 1. On January 11, 1912, she had another attack, which affected the area shown in Figs. 1 and 11. In June, 1912, a third attack, as shown in Figs. III, on right hand. Figs. III and IV show a pigmentation of the area affected in January. In July, 1912, she had the dermatitis on the left hand. The winter attacks were severe, while the summer attacks were represented by a slight dermatitis with no constitutional symptoms..

The cases of Group VIII had not eaten cottonseed oil products for the following number of months before the last attack.

| | | | | | | | | | |
|---|---|---|---|---|---|---|---|---|---|
| Case | No. | 66, | no | cotton | seed | oil | in | 7 | months; |
| " | " | 67, | " | " | " | " | " | 5 | " |
| " | " | 68, | " | " | " | " | " | 9 | " |
| " | " | 69, | " | " | " | " | " | 9 | " |
| " | " | 70, | " | " | " | " | " | 9 | " |
| " | " | 71, | " | " | " | " | " | 8 | " |
| " | " | 72, | " | " | " | " | " | 8 | " |

| | | | | | | | | | |
|---|---|---|---|---|---|---|---|---|---|
| Case | No. | 73, | no | cotton | seed | oil | in | 7 | montths |
| " | " | 74, | " | " | " | " | " | 7 | " |
| " | " | 96, | " | " | " | " | " | 9 | " |
| " | " | 97, | " | " | " | " | " | 9 | " |
| " | " | 98, | " | " | " | " | " | 10 | " |
| " | " | 99, | " | " | " | " | " | 11 | " |

If time permitted, we could show that the severity of the last attack and the state of health of the pellagrin is in proportion to the length of the period that oil had been discarded and the extent of emaciation present when it was discarded.

*Age.* All ages appear among these cases. Case No. 100 is a child, eight months old, born in Atlanta, and had not been out of the city. His father was a pellagrin and his mother showed some symptoms, but was in fair health. The child, of course, does not eat cottonseed oil products, but we only need to mention that the infant comes into the world with a store of fat and an excess of lecithin which is probably not used up in the first months of extrauterine life, and that the fatty tissues of its body comes from, and is influenced by the food of the mother.

Of the one hundred cases, thirteen are under twenty years, seven are over forty.

*Sex.* Seventy-nine out of the one hundred are females. It is interesting to note that at the extremes of life this great preponderance of females is not so marked. Of nine under fifteen years of age, five are girls, while of those over fifty years of age, nine are women and five are men. The preponderance of gastro-intestinal disorders among women in this section, together with disturbances incident to female sexual life, may account for the difference in incidence in the sexes. Sixty-three per cent. of gastro-intestinal cases coming for treatment are women. It cannot be doubted that chronic diseases, such as some female disorders, predispose to pellagra. It is a notable fact that pellagra in the otherwise healthy is comparatively rare.

*Residence.* In designating the residence, no cases are classified as urban unless they have lived in the city for two years when the first attack of dermatitis appeared. Those living in towns of less than three thousand inhabitants are classified as rural.

The table shows sixty-two cases of urban residence and thirty-eight of rural residence. These cases have made no material change in residence during the years of observation.

The incidence of pellagra among farmers is entirely limited to those who are using cottonseed oil. Much more corn products are consumed per individual by rural inhabitants and the western product is as much in use among them as among urban inhabitants.

*Seasonal incidence.* The one hundred cases have had 170 attacks of dermatitis the months of which could be assertained. The month of incidence is as follows:

| | | | |
|---|---|---|---|
| January, | 2 | July, | 31 |
| February, | 2 | August, | 8 |
| March, | 17 | September, | 4 |
| April, | 24 | October, | 2 |
| May, | 39 | November, | 0 |
| June, | 40 | December, | 1 |

Attacks occurring in the winter are not rare and we must admit other exciting causes than seasonal influences. Time does not permit full discussion, which would show that this theory is in harmony with the history, seasonal incidence, geographical distribution, pathology and course of disease.

401-3 Empire Life Building.

## Figures and Tables

**FIGURE I. f1:**
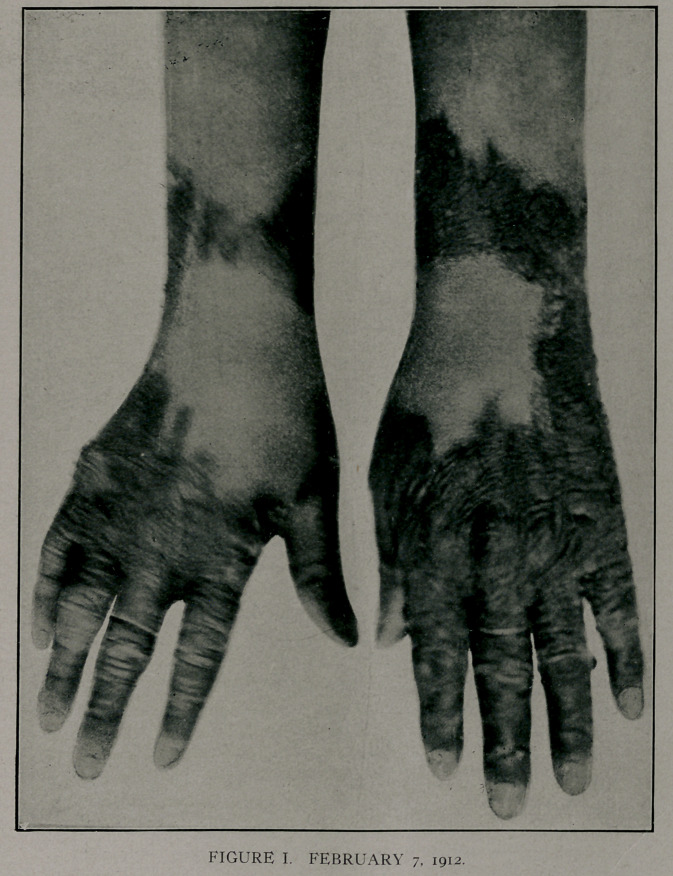


**FIGURE II. f2:**
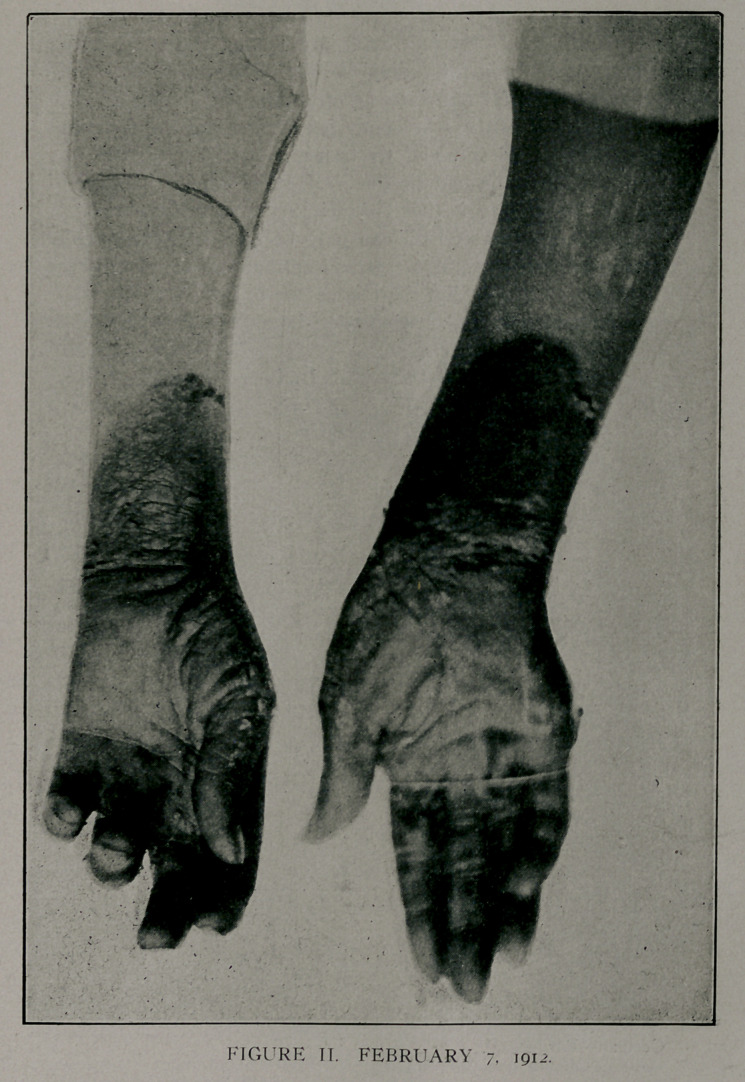


**FIGURE III. f3:**
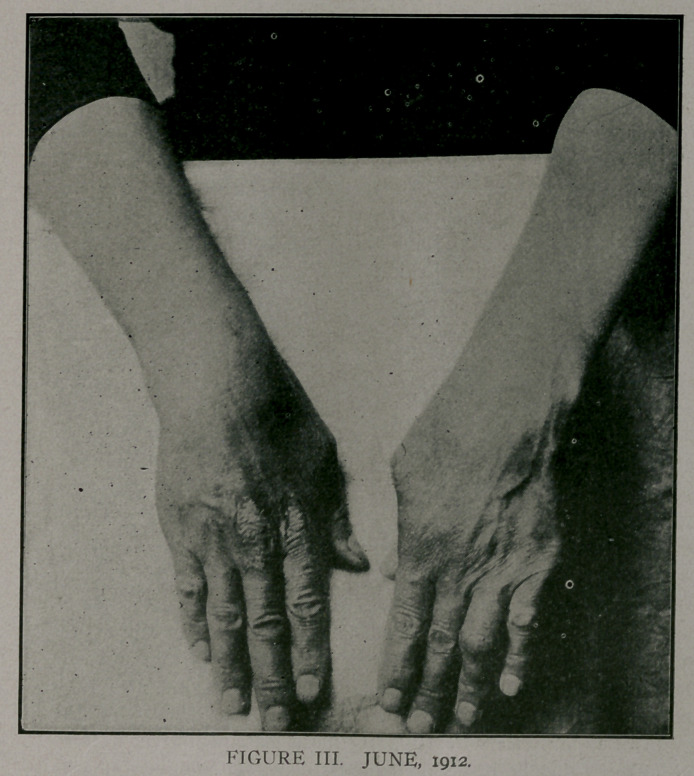


**FIGURE IV. f4:**